# Supplementary material for: Duodenal Biopsy Audit: Relative Frequency of Diagnoses, Key Words on Request Forms Indicating Severe Pathology, and Potential Diagnoses for Intraepithelial Lymphocytosis, as a Foundation for Developing Artificial Intelligence Diagnostic Approaches
Source: Diagnostics (Basel). 2025 Jun 11;15(12):1483. doi: 10.3390/diagnostics15121483 (PMC12191449; doi:10.3390/diagnostics15121483)
Supplement: Supplementary file 1 [file diagnostics-15-01483-s001.zip › diagnostics-3582171-supplementary.pdf]

diagnostics

MDPI

Article

Duodenal Biopsy Audit: Relative Frequency of Diagnoses, Key Words on Request Forms Indicating Severe Pathology, and Potential Diagnoses for Intraepithelial Lymphocytosis, as a Foundation for Developing Artificial Intelligence Diagnostic Approaches

Vrinda Shenoy [1], Jessica L. James [1], Amelia B. Williams-Walker [1], Nasyen P. R. Madhan Mohan [1], Kim N. Luu Hoang [1], Josephine Williams [2], Florian Jaeckle [1,3], Shelley C. Evans [1] and Elizabeth J. Soilleux [1,3,*]

[1] Department of Pathology, University of Cambridge, Tennis Court Road, Cambridge CB2 1QP, Cambridgeshire, UK; vs534@cantab.ac.uk (V.S.); jlj47@cam.ac.uk (J.L.J.); aw2000@cam.ac.uk (A.B.W.-W.); nprm2@cam.ac.uk (N.P.R.M.M.); knl25@cam.ac.uk (K.N.L.H.); sce30@cam.ac.uk (S.C.E.); fj286@cam.ac.uk (F.J.)

[2] Human Research Tissue Bank, Cambridge University Hospitals NHS Foundation Trust, Cambridge CB2 0QQ, Cambridgeshire, UK; josephine.williams15@nhs.net

[3] Lyzeum Ltd., Salisbury House, Station Road, Cambridge CB1 2LA, Cambridgeshire, UK

* Correspondence: ejs17@cam.ac.uk

Academic Editor: 29 May 2025

Received: 27 March 2025
Revised: 29 May 2025s
Accepted: 9 June 2025
Published: date

Citation: Shenoy, V.; James, J.L.; Williams-Walker, A.B.; Madhan Mohan, N.P.R.; Luu Hoang, K.N.; Williams, J.; Jaeckle, F.; Evans, S.C.; Soilleux, E.J. Duodenal Biopsy Audit: Relative Frequency of Diagnoses, Key Words on Request Forms Indicating Severe Pathology, and Potential Diagnoses for Intraepithelial Lymphocytosis, as a Foundation for Developing Diagnostic Artificial Intelligence Approaches. *Diagnostics* **2025**, *15*, x. https://doi.org/10.3390/xxxxx

Copyright: © 2025 by the authors. Submitted for possible open access publication under the terms and conditions of the Creative Commons Attribution (CC BY) license (https://creativecommons.org/licenses/by/4.0/).

Abstract: Background/Objectives: Understanding the diagnostic landscape is essential prior to developing artificial intelligence (AI)-based diagnostic strategies for automating the diagnosis of duodenal biopsies. This study aims to (1) determine the frequencies of different diagnoses seen in endoscopic duodenal biopsies in a large, tertiary referral centre; (2) identify key words on histopathology request forms that could indicate that a biopsy may contain a serious pathology and should not be diagnosed by an AI system; and (3) investigate the proportion of cases described as showing "intraepithelial lymphocytosis" that might be coeliac disease. Methods: To achieve this, we audited 18 months' worth of duodenal biopsy reports in our centre. Results: A total of 6245 duodenal biopsies were identified, of which 73.76% were normal and at least 8.84% fell within the spectrum of coeliac disease. Additionally, 6.47% were classified as showing non-specific inflammation, 1.86% were adenomas, 0.45% were carcinomas, 0.06% were neuroendocrine tumours, 0.10% were lymphomas, and 0.03% were cases of flat dysplasia, giving a total of 0.64% of dysplastic or malignant diagnoses. Rarer diagnoses included ulceration, *Helicobacter pylori* infection, giardiasis, lymphangiectasia, transplant rejection, and lymphoma. Furthermore, 227 biopsies (3.63%) showed isolated intraepithelial lymphocytosis, of which 33 cases (14.5%) gave an overall clinicopathological picture of coeliac disease. Conclusions: We present the first long-term audit of all endoscopic duodenal biopsies received by the histopathology department of a tertiary-care facility. The results indicate that a fully automated diagnostic histopathology reporting system able to identify normal duodenal biopsies and biopsies within the spectrum of coeliac disease-associated enteropathy could decrease pathologists' endoscopic duodenal biopsy workload by up to 80%.

Keywords: coeliac disease, audit, duodenal biopsy, gluten sensitivity.

1. Supplementary Materials

A. HLA typing for Isolated Intraepithelial Lymphocytosis Cases

HLA typing was performed on 21 of the 227 patients with isolated intraepithelial lymphocytosis, revealing that 14 (66.7%) carried at least one coeliac disease risk allele (HLA-

DQ2.5, HLA-DQ2.2, or HLA-DQ8) (Table S1). Of these 14 cases, 4 either received a definitive CeD diagnosis or exhibited blood test results indicative of CeD. Although a CeD-associated HLA type is valuable when combined with a strong clinical history in pediatric clinical practice[1], it should not be considered conclusive evidence of the disease. This is underscored by data from the UK Biobank, where 48% of individuals without CeD carry these risk alleles compared to 98% of CeD patients (Table S1). Assuming a CeD prevalence of 1%, in the absence or any other patient information (e.g. symptoms, blood tests, biopsies, previous medical or family history), the chance of having CeD increases from 1% to 1.6% if a patient has a CeD risk allele and decreases to 0.18% if they do not. However, it is important to note that the presence of symptoms, blood tests, or biopsy results may alter these probabilities.

Table S1: Coeliac disease-associated HLA haplotypes and their carrier frequencies among the audited patients with isolated intraepithelial lymphocytosis (n = 21, the total number of patients with available HLA test data), compared to the data examined from 12,227 patients' genome-wide genotypes derived from the UK Biobank, using HLA imputation values provided by the UK Biobank. 98% of CeD patients (n = 1281) and 58.1% of control participants (n = 10 964) in the 200,000 exome UK Biobank dataset have a CeD risk allele. The highlighted column includes patients with no CeD risk alleles. The absence of these alleles can be used to significantly decrease the likelihood of CeD.

***HLA* genotypes**	*HLA-DQ2.5*	*HLA-DQ8*	*HLA-DQ2.2*	*HLA-DQ2.5 & DQ8*	*HLA-DQ2.5 & DQ2.2*	*HLA-DQ8 & DQ2.2*	**CeD risk HLA types**	**Other HLA types**	**Total**
**Biobank CeD participants**	777 (61%)	79 (6%)	132 (10%)	56 (4%)	191 (15%)	20 (2%)	1255 (98%)	26 (2%)	1281
**Biobank Controls**	2,411 (22%)	1,629 (15%)	1,581 (14%)	275 (3%)	267 (2%)	202 (2%)	6365 (58%)	4,599 (42%)	10,964
**Isolated intraepithelial lymphocytosis cohort**	8 (38%)	1 (5%)	5 (24%)	0	0	0	14 (67%)	7 (33%)	21

*HLA – Human Leukocyte Antigen

2. Supplementary Results

Table S2: Definitions of nomenclature used in Tables 1, 2 and 5, and Figure 2.

Classification	Explanation of terminology

**Active coeliac disease**	Damage to the small intestine due to ongoing ingestion of gluten (a protein found in wheat, barley, and rye), histologically characterized in a small intestinal biopsy by villous atrophy (shortening of the finger-like processes in the small intestinal lining), crypt hyperplasia (enlargement and elongation of the gland-like structures between the intestinal villi), and increased numbers intraepithelial lymphocytes (a type of white blood cell that can be found in the surface layer of the small intestinal wall).
**Coeliac disease on GFD: normal biopsy**	Biopsy from a patient known to have coeliac disease, who is eating a gluten-free diet. The biopsy appears normal.
**Coeliac disease on GFD: partially recovered biopsy**	Biopsy from a patient known to have coeliac disease, who is eating a gluten-free diet. The biopsy shows features intermediate between normal and active coeliac disease.
**Refractory coeliac disease**	Patient with coeliac disease, in whom symptoms and intestinal damage persist or recur despite strict adherence to a gluten-free diet for more than 12 months.
**Isolated villous atrophy**	Shortening of the finger-like processes in the small intestinal lining, without other changes of coeliac disease.
**Villous atrophy + raised IELs**	Shortening of the finger-like processes in the small intestinal lining, without crypt hyperplasia (enlargement and elongation of the gland-like structures between the intestinal villi), but with increased numbers intraepithelial lymphocytes (a type of white blood cell that can be found in the surface layer of the small intestinal wall).
**Gluten sensitive enteropathy**	A term used interchangeably with coeliac disease.
**Isolated intraepithelial lymphocytosis**	Increased numbers intraepithelial lymphocytes (a type of white blood cell that can be found in the surface layer of the small intestinal wall), without other histological changes of active coeliac disease.
**Non-specific inflammation**	Increased numbers of white blood cells. Most pathologists would not use this term to describe increased numbers of intraepithelial lymphocytes (intraepithelial lymphocytosis).
**Adenoma**	A tumour (growth) of the intestinal epithelium, in which the abnormal epithelial cells do not invade into structures beyond the epithelial layer.

**Carcinoma**	A tumour (growth) of the intestinal epithelium, in which the abnormal epithelial cells invade into structures beyond the epithelial layer (cancer).
**Lymphoma**	A cancer of a particular type of white blood cells.
**Lipoma**	A benign tumour of fat cells.
**Neuroendocrine tumour**	A malignant tumour derived from duodenal epithelium, which recapitulates the differentiation of a specialist epithelial cell type.
**Flat dysplasia**	Histologically abnormal (pre-cancerous) epithelial cells in the duodenal lining which do not form a visible lump or growth and which do not invade into structures beyond the epithelium.
**Giardiasis**	Infection with the small intestinal parasite, Giardia. The parasites are visible histologically.
***Helicobacter pylori* infection**	Infection, generally in the stomach, but rarely in the duodenum, with the bacterium, *Helicobacter pylori.* The bacteria are visible histologically.
**Viral enteropathy**	Viral infection, leading to subtle, but characteristic changes in the epithelium.
**Focal gastric heterotopia**	Some of the epithelium in the duodenum has changed its appearance to look like stomach rather than duodenal epithelium.
**Gastric metaplasia**	Some of the epithelium in the duodenum has changed its appearance to look like stomach rather than duodenal epithelium.
**Ulceration**	Loss of epithelial surface tissue and some tissue from deeper layers of the duodenal wall, often associated with some inflammation. Deeper than an erosion.
**Lymphangiectasia**	Dilatation of the lymphatic vessels in the duodenal wall.
**Erosion**	A superficial loss of the surface layer (the epithelium and a very small amount of underlying connective tissue that make up the mucosa).
**Regenerative changes**	Healing or repair processes in the lining of the duodenum.
**Hyperplasia**	An increase in the number of cells in a tissue or organ, resulting in its enlargement. This can sometimes occur in response to a previous or ongoing injury.
**Crohn's disease**	Type of inflammatory bowel disease, i.e., a chronic inflammatory condition, which has relatively characteristic histological changes.
**Drug-induced enteropathy**	Inflammatory changes and sometimes villous atrophy occurring while a patient takes a specific

	medication, with duodenal biopsy appearances reverting to normal when the medication is discontinued.
**Lymphoid aggregates**	Clusters of a type of white blood cell/ inflammatory cell known as a lymphocyte present in the biopsy.
**Granuloma**	Inflammatory process directed towards a foreign bodies, medications, infections, Crohn's disease etc, in which white blood cells/ inflammatory cells called macrophages are grouped together, with a small amount of fibrosis (scarring) around them.
**Transplant**	Miscellaneous complications of transplantation, such as rejection (if biopsying the duodenum of a donor small intestine in an intestinal transplant recipient).
**Graft versus host disease (GvHD)**	Inflammatory process that occurs in bone marrow transplant recipients, in which lymphocytes derived from the bone marrow donor attack recipient tissues, including the small intestinal epithelium.
**Amyloidosis**	Disease in which aggregates of abnormal protein are laid down in various parts of the body, including (rarely) parts of the intestine.
**Thrombus**	Blood clotting occurring in a blood vessel
**Pseudolipomatosis**	Small gas bubbles are seen in the biopsy, most likely caused by air introduced into the duodenal mucosa during endoscopy.
**Spindle cell tumour**	
**Pseudomelanosis**	
**Gangliocytic paraganglioma**	Rare neural tumour of the duodenum, which is generally benign.
**Common variable immunodeficiency**	Condition in which the immune system does not respond adequately to some or all infectious agents, which can be associated with infections that are rarely seen in individuals with a normal immune system.
Proton pump inhibitor use	Use of a medication associated with treatment of excess stomach acid, e.g., omeprazole, lansoprazole.
**Non-steroidal anti-inflammatory drug** (NSAID) use	Use of a medications of the aspirin class.
Irritable bowel syndrome	Condition in which patients suffer stomach cramps, bloating, diarrhoea and constipation. Coeliac disease and inflammatory bowel disease can also cause these symptoms and must be excluded prior to making the diagnosis.
Hypothyroidism	Insufficient production of thyroid hormone by the thyroid gland, most frequently due to autoimmune disease.
Graves' disease	Excess production of thyroid hormone by the thyroid gland, most frequently due to autoimmune disease.

Microscopic colitis	A relatively mild type of inflammatory bowel disease that can be associated with autoimmune conditions, including coeliac disease, and medications including proton pump inhibitors and non-steroidal anti-inflammatory drugs.
**Pernicious anaemia**	An autoimmune disease of the stomach that prevents proper absorption of vitamin B12, which goes on to cause anaemia (insufficient quantities of red blood cells).
Crohn's disease	A type of inflammatory bowel disease.
Rheumatoid arthritis	An autoimmune disease which causes arthritis.
Ankylosing spondylitis	An autoimmune disease which causes arthritis.
Gastrointestinal adenocarcinoma	A type of carcinoma (see above) that forms glands.
Ulcerative colitis	A type of inflammatory bowel disease.
Seronegative spondyloarthropathy	An autoimmune/ inflammatory disease which causes arthritis.
Addison's disease	An autoimmune disease which causes insufficient production of the adrenal hormones, cortisol and aldosterone, leading to problems maintaining fluid balance and blood pressure, and a less effective response to physiological stress.

References

(60) *How Should I Assess a Person with Suspected Coeliac Disease?*; NICE. https://cks.nice.org.uk/topics/coeliac-disease/diagnosis/assessment/#:~:text=Consider%20checking%20serum%20IgG%20EMA,care%20to%20diagnose%20coeliac%20disease. (accessed 2025-02-19).
